# Recalibrating the calcium trap in amino acid carboxyl groups *via* classical molecular dynamics simulations†

**DOI:** 10.1039/d2cp02879d

**Published:** 2022-12-05

**Authors:** Janou A. Koskamp, Sergio E. Ruiz Hernandez, Nora H. de Leeuw, Mariette Wolthers

**Affiliations:** a Department of Earth Sciences, Utrecht University 3584 CB Utrecht The Netherlands j.a.koskamp@uu.nl s.e.ruizhernandez@uu.nl n.h.deleeuw@uu.nl m.wolthers@uu.nl +31302535042; b School of Chemistry, University of Leeds Leeds LS2 9JT UK n.h.deleeuw@leeds.ac.uk

## Abstract

In order to use classical molecular dynamics to complement experiments accurately, it is important to use robust descriptions of the system. The interactions between biomolecules, like aspartic and glutamic acid, and dissolved ions are often studied using standard biomolecular force-fields, where the interactions between biomolecules and cations are often not parameterized explicitly. In this study, we have employed metadynamics simulations to investigate different interactions of Ca with aspartic and glutamic acid and constructed the free energy profiles of Ca^2+^–carboxylate association. Starting from a generally accepted, AMBER-based force field, the association was substantially over and under-estimated, depending on the choice of water model (TIP3P and SPC/fw, respectively). To rectify this discrepancy, we have replaced the default calcium parameters. Additionally, we modified the *σ*_*ij*_ value in the hetero-atomic Lennard-Jones interaction by 0.5% to further improve the interaction between Ca and carboxylate, based on comparison with the experimentally determined association constant for Ca with the carboxylate group of l-aspartic acid. The corrected description retrieved the structural properties of the ion pair in agreement with the original biomolecule – Ca^2+^ interaction in AMBER, whilst also producing an association constant comparable to experimental observations. This refined force field was then used to investigate the interactions between amino acids, calcium and carbonate ions during biogenic and biomimetic calcium carbonate mineralisation.

## Abstract 2

In order to use classical molecular dynamics to complement experiments accurately, it is important to use robust descriptions of the system.

## Graphical abstract

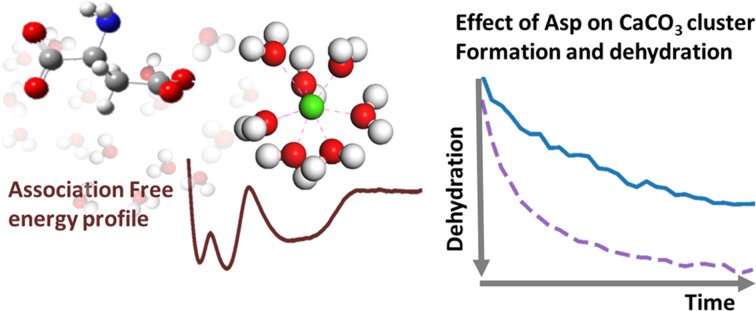

## Introduction

Calcium is an important ion in many biological processes, for example interacting with biomolecules to regulate enzyme activity.^1^ It is also an important building block in biominerals, *e.g.* in the bone mineral apatite and in CaCO_3_ minerals to create for example the shells of molluscs, eggs and corals (*e.g.* ref. 2,3). The conditions under which such biominerals are formed are complex and some of the biomineralization mechanisms remain unresolved. Atomistic computational approaches can be employed to provide detailed insight into the structure and dynamics between calcium ions and complex biomolecules that are considered to play an important role in biomineralization (*e.g.* ref. 4–8).

Biomolecules are complex, usually flexible, systems containing many atoms. The treatment of such many-electrons systems using pure *ab initio* or density functional theory (DFT) methods is highly compute-intensive and, in some cases, simply unachievable. Therefore, computationally less demanding classical atomistic simulation techniques are widely used to simulate proteins and large peptides and their interactions with other systems. Multiple force fields and software packages have been developed to accurately describe a range of biomolecules. For example, AMBER,^9^ CHARMM^10^ and GROMOS^11^ are software packages with their own set of biomolecular force fields and all are widely used to perform molecular dynamics simulations (MD). Over the years, the force fields have been improved and updated alongside developments in experimental techniques and insights gained from *ab initio* calculations.^12–17^

Generally, biomineralization is thought to be guided by proteins that are rich in glutamic and aspartic acid that trap calcium.^18–20^ However, in classical MD this calcium-trapping is not observed for these amino acids, nor in small aspartic acid–based biomolecules.^4,21^ This observation is in contrast with conclusions from experiments, where biomolecules bind Ca,^22–25^ and *ab initio* (DFT) calculations with implicit water, which have reported a shorter distance between oxygen of the carboxyl group in the amino acid and Ca than between carboxyl and carbonate.^22^ To obtain meaningful insights into the calcium trapping behaviour of these specific amino acids and their impact on CaCO_3_ formation, it is important to address why classical computational outcomes differ from experimental work.

In atomistic simulations, it is crucial to achieve an accurate description of the free energy surface (FES) of the system.^26^ Although *ab initio* calculations commonly provide the most reliable results, the high computational costs make them less suitable to calculate the FES for large biosystems.^26^ In these large systems the convergence requires very prolonged simulations, necessary to generate accurate data. It is worth noting that recently an *ab initio* investigation verified results obtained with classical MD, despite inherent features of the latter such as fixed-charge force-fields and a lack of accounting polarizability and nuclear quantum effects.^27^

By using a force field, as in MD approaches, the FES is mimicked and thereby physical insights are acquired. The derivation of such force fields can be achieved through a number of approaches, resulting in multiple ways to describe the same system. In commonly used force fields, the non-bonding interactions are obtained using one of the Lorentz–Berthelot mixing rules and in many cases the parameters for the non-biological interactions in the systems (*i.e.* between ions, water, *etc.*) are transferred from other published parameters, which in some cases may lead to an unphysical or incorrect description of the FES.^28^

In this work, we have used well-tempered metadynamics simulations to investigate the interaction of Ca with the carboxylate oxygen (O_carboxylate_ or OD) in glutamic and aspartic acid dissolved in water. We have compared the binding energies of our AMBER-based force field with experimental data. Using different Ca descriptions, our results show that the standard AMBER parameters combined with a Ca description reported previously^29^ either under- or over-estimate the Ca–O_carboxylate_ binding energy. We therefore propose a refinement of the Ca–O_carboxylate_ Lennard-Jones (LJ) parameters from the SPC/fw water model and a Ca description based on previously reported parameters^29^ used in combination with AMBER-based biomolecules with carboxyl groups. Using this refinement, MD simulations show the impact of aspartic acid on growing amorphous calcium carbonate clusters, with the most striking result the inhibition of dehydration of the formed ACC.

## Methods

For the calculations of the free energies, and the refinement of the interatomic potential parameters for dissolved calcium and the carboxylate-oxygen interaction in specific biomolecules, we have created two different simulation cells, both containing 2163 water molecules and one Ca^2+^. The first cell contains an l-aspartic acid (l-Asp) molecule, and the second cell contains a glutamic acid molecule. The representation of the biomolecules corresponds to a pH condition ∼10. Using the refined force field parameters, we studied the impact of aspartic acid on the spinodal decomposition of a CaCO_3_ rich solution. We placed one enantiomers (l or d) of aspartic acid in a box with 15831 water molecules, and 255 Ca^2+^ and 255 CO_3_^2−^ ions randomly distributed. The systems contained either one l-Asp, one d-Asp or nothing (pure). In the systems with biomolecules, two Na^2+^ were added as background electrolytes to charge balance the 2e-overall charge of the biomolecule. We created four different starting configurations, to enhance the statistics of the results, and a similarity analysis between the initial configurations was conducted to quantify the differences in ion distributions using the Tanimoto distance.^30–32^ For the Tanimoto distance, the vectors (configuration A and B, eqn (1)) were ordered according to the distance to the center of mass (COM) of the biomolecule, whereas the pure systems were created by removing l-Asp from the starting configurations. The vectors contain, first, the distance between the atom closest to the COM of the biomolecule and the other atoms in the CaCO_3_ cluster, followed by the distances between the atom closest to this first atom and the other atoms and so on. The Tanimoto distance was calculated and only random ion-configurations with a Tanimoto distance of less than 0.7 were used to guarantee that the starting configurations were different.1
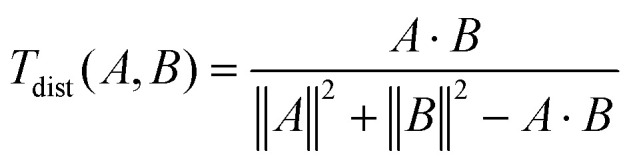
The LAMMPS (Large-scale Atomic/Molecular Massively Parallel Simulator) code was used to perform the MD simulations.^33^ The simulation temperature and pressure were kept at 300 K and 1 atm, respectively, in the *NPT* ensemble, using 0.1 and 1 ps relaxation times for the Nosé–Hoover^34,35^ thermostat and barostat, respectively. The equations of motion were integrated using the velocity-Verlet algorithm^34,35^ with a time step of 1 fs. Every system was simulated for 30 ns with a 1 ns equilibration period.

The description of the biomolecules was taken from the AMBER force field using the leap program to generate all the structures and extract the topology and interaction parameters.^36^ The amino acids were C-terminated and capped with a proton on the nitrogen to produce the final structure (Fig. S1, ESI†). The Ca^2+^ and CO_3_^2−^ ions were simulated using the parameters from ref. 37 and the parameters for the water are those of the SPC/fw force field,^38^ which we have chosen given the validated CaCO_3_ interactions^29,39^ and because the TIP3P model (incorporated in AMBER) has a diffusion coefficient which is twice the experimental value.^40^

We used the Lorentz–Berthelot rules (for consistency with AMBER, where the same rules have been applied^36^) to create the Lennard-Jones potentials between the Ca and the atoms in the biomolecule.^41,42^ Several parameter sets (*ε*_*ii*_ and *σ*_*ii*_) for the description of Ca using Lennard-Jones potentials can be found in the literature, Table 1. Out of the several sets, we tested three different sets of parameters for Ca in combination with the biomolecules and labelled them Set_1, Set_2 and Set_3 (Table 1 and in the (ESI†) Table S1 until Table S3). For completeness, the Ca parameters of Set_1^9^ were tested using both the SPC/fw and AMBER's original TIP3P water models (ESI,† Table S1). The Set_2 Ca parameters were based on a previously reported force field,^29^ which we extracted from the O_water_–O_water_ and O_water_–Ca interaction parameters (ESI,† Table S2). Set_3 was created by modifying Set_2 to refine the interactions for Ca–amino acid and the corresponding energy profile (ESI,† Table S3). This was done by changing both *ε* and *σ* parameters;^28,43,44^ in this case *σ*_Ca_Set3_ and *ε*_Ca_Set3_ were taken as the average of Set_2 and the parameters of O_carboxylate_. In the LJ-interaction, *σ* alters the distance of the well and thus the equilibrium distance between the ion pair, which may also change the depth of the well. We increased the *σ* LJ-parameter for Ca^2+^–O_carboxylate_ stepwise up to +2.0% (see Table 3) to decrease the well depth to match experimental observations, in a similar approach to the method described in. ref. 28

**Table 1:** Comparison of different Ca Lennard-Jones potential parameters to be combined with the biomolecule force field

Ca^2+^	Acronym	*ε*_*ii*_ (eV)	*σ*_*ii*_ (Å)	*A*_*ii*_	*B*_*ii*_	*A*_*ii*_/*B*_*ii*_
Hornak, 2006^9^	Set_1	0.019937830	3.426200	208685	129	1618
Mamatkulov, 2013^50^ AMBER		0.019899403	3.050000	51582	64	805
Aqvist, 1990^51^		0.019692117	2.410000	3024	15	196
Wang, 2008^52^		0.011457217	2.789015	10152	22	471
Mamatkulov, 2013^50^		0.009742416	2.410000	1496	8	196
Martinek, 2017^53,54^		0.005256800	2.819600	5309	11	502
Beglov, 1994^55^ CHARMM		0.005182136	2.430000	879	4	206
Dang, 1995^56^		0.004336300	2.895000	6011	10	589
Shen^57^		0.003316575	2.450000	621	3	216
Babu and Lim, 2006^58^		0.001243713	3.250000	6908	6	1178
This work (based on ref. 39)	Set_2	0.000133902	3.534508	2036	1	1950
Adjusted Set_2	Set_3	0.001104240	3.247210	6071	5	1172

The free energy profiles were constructed using well-tempered metadynamics simulations for every modified set of interactions. The PLUMED 2.5.3^45^ plug-in for LAMMPS was used to perform all free energy calculations. The collective variable (CV) was defined as the distance between Ca and the central carbon of the carboxylate group of the biomolecule. Gaussian hills were deposited every 10 ps with a hill height of 1.0 kJ mol^−1^ and a width of 0.02 nm. The bias factor was set at 10. Since Ca is considered fully dissolved at approximately 1 nm, we explored the CV up to 1.6 nm distance, and we placed an upper wall at 1.6 nm with a force constant (*κ*) of 2000. The total simulation time was 100 ns per metadynamics simulation to achieve convergence. The obtained free energy profiles were normalized by setting the asymptotic region to zero. The average energy of the CV distance from 1 to 1.4 nm was set to zero and the correction value was subtracted from the energy profile.

The comparison of the free energy profiles from the metadynamics simulations with the experimentally obtained association constant (*K*_a_) was achieved through predicting the *K*_a_ from the profiles. *K*_a_ is a stability constant, in the literature also referred to as the formation or binding constant, and in terms of activities, *K*_a_ is defined as:2
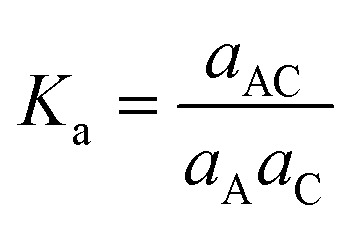
where *a*_AC_ is the activity of the anion (ligand)–cation complex, while *a*_A_ and *a*_C_ are the activities of the free anions and cations in solution. The association constant of calcium to the ligand as seen in experiments covers a multistep process.^46^ Upon association, the calcium approaches the ligand, expressing multiple metastable association structures: the solvent-separated ion pair (SSIP) describing a pair of single ions, solvent-shared ion pairs (SIP) and the contact ion pair (CIP). It is therefore important to consider all states of association when processing MD results. Another way of expressing *K*_a_ is:^47^3
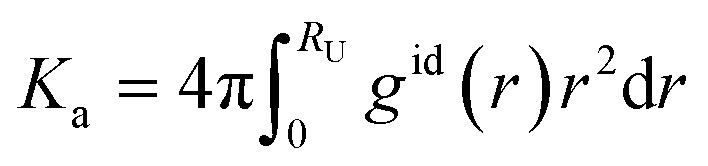
with *g*^id^(*r*) as the radial distribution function (RDF) of the ligand and the cation, *r* the radial distance and *R*_U_ the upper limit corresponding to the distance between the cation and the ligand in the dissociated state. The dissociated state is defined as the first local minimum in the *g*^id^(*r*) beyond the SIP maximum (around 0.8 nm). To obtain the *K*_a_ from our own MD, we converted the energy profile into a radial distribution function *g*^id^(*r*), as we assumed an infinite dilution of the ion pair:4
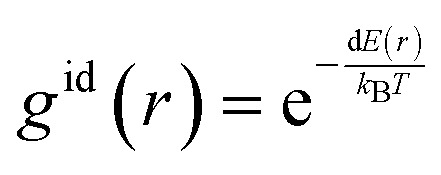
where d*E*(*r*) is the free energy profile in kJ mol^−1^ along the CV distance, *k*_B_ the Boltzmann constant (kJ K^−1^) and *T* the temperature in K. The obtained *K*_a_ could then be converted into the Gibbs free energy (d*G*):

5 d*G*_association_ = −*RT *ln(*K*_a_)

where *R* is the gas constant. The advantage of this method over computing the d*G*_association_ as the difference between the free energy minimum and the asymptotic limit for long-range separation, is that now the width and depth of the local wells are accounted for and thus the whole profile carries more weight compared to taking a single minimum and an asymptotic maximum.

To investigate the impact of our modified Ca–ligand interaction from the AMBER-based force field, we analysed the formation of an amorphous calcium carbonate (ACC) cluster in the presence of l- or d-aspartic acid. An in-house python script was used to calculate the number of clusters and their sizes in terms of ions over the course of the simulation. An ion was considered part of a cluster if the distance to another ion in the cluster was less than the distance of the first coordination shell of the two species. For example, the maximum distance between Ca^2+^ and O_c_ was set to 0.34 nm (based on the first minimum in the Ca–O_c_ RDF). We averaged the cluster size distributions of the duplicates to follow the evolution in time. To study the possibility of the presence of water in and around the clusters, the solvent accessible surface area (SASA) was computed over the full trajectory to follow the change with time, using the Mdtraj^48^ module in the Anaconda Python distribution. The method consisted of running the Shrake and Rupley^49^ algorithm to explore the area around all Ca^2+^ and CO_3_^2−^ ions with a probe of the size of a water molecule (radius = 0.14 nm). The local density of ACC (Ca^2+^ and CO_3_^2−^) was calculated from the integral of the radial distribution function until 0.9 nm divided by the volume corresponding to that radius. Several radial distribution functions were calculated between the different ions and functional groups, where the RDFs were calculated from the final 10 ns of each simulation.

## Results and discussion

### Calcium description

The description of Ca^2+^ in interatomic Lennard-Jones potentials for MD varies over different studies (Table 1). As can be seen from Table 1, *ε* varies by more than one order of magnitude while the maximum difference in *σ* is approximately 0.1 nm. The intrinsic Pauli repulsion is described by the *A* parameter (*A* = 4π*εσ*^12^), while the attraction between two Ca ions is described by the *B* parameter (*B* = 4π*εσ*^6^). For the highest and the lowest value of *ε*, the *A* parameter of Set_1^9^ is 2.0 orders of magnitude larger than the Ca repulsion of Set_2. The attraction that compensates this repulsion, was also a 2.1 order of magnitude larger, which resulted in a similar balance between repulsion and attraction as seen in the *A*/*B* values. In the continuing sections, we have compared the two most extreme descriptions for Ca, *i.e.*, Set_1 and Set_2, and the proposed alternative description, Set_3.

### Free energy of association

**Fig. 1:**
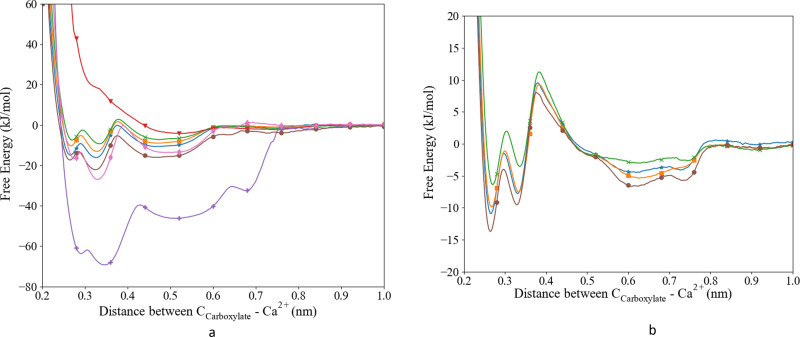
Free energy profiles for the interaction between Ca^2+^ and the O_carboxyl_. (a) l-Asp and (b) glutamic acid. Set_1_TIP3P_ (purple; 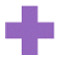), Set_1_SPC/fw_ (red; 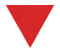), Set_2 (pink; 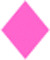), Set_3_unmodified_ (brown; 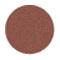), Set_3_*σ*+0.5%*σ*_ (blue; 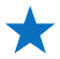), Set_3_*σ*+1.0%*σ*_ (orange; 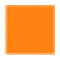), Set_3_*σ*+2.0%*σ*_ (green; 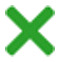).

The potential parameters of Ca were combined with the parameters of the biomolecule to describe the interaction with the aspartic acid and glutamic acid as used in our MD. To evaluate and improve the Ca^2+^–O_carboxylate_ interaction, the MD results were reviewed considering the experimental *K*_a_ and the coordination distance obtained from *ab initio* MD. As a starting point we constructed the free energy profiles using the unmodified Set_1, Set_2 and Set_3 interactions, Fig. 1a. We combined the parameters from Set_1 with TIP3P and SPC/fw water. The combination with TIP3P resulted in the deepest well in the energy profile compared to all other parameters published here. The well ends at 0.78 nm and has four local minima at 0.290, 0.346, 0.513 and 0.677 nm. The minima correspond to different Ca–ligand–water structures, *i.e.*, the first and second minima correspond to a CIP that was split in a bidentate and a monodentate configuration. In the bidentate (biCIP) configuration, Ca was coordinated to both oxygens in the carboxylate group, while in the monodentate (monoCIP) configuration, Ca was only coordinated to one of the oxygens. The third and the fourth minima resemble SIP and SSIP, respectively.

From the free energy profile, we extracted the largest Δ*G*_a_ (−62.3 kJ mol^−1^) and *K*_a_ (7.07 × 10^10^) compared to the other parameter sets and literature values (Table 2). The coordination distance between Ca and O_carboxylate_ was slightly longer than observed experimentally. The TIP3P water model was then replaced by the SPC/fw water model in combination with Set_1, since this water model has been found to behave in a more realistic manner.^40^ However, the corresponding free energy profile of this system did not show clear minima Fig. 1a. The global minimum was at 0.546 nm which indicated a long Ca–O_carboxylate_ distance of 0.466 nm. The shallow minimum in the energy profile led to the lowest observed *K*_a_ (Table 2). Neither water models in combination with the parameters from Set_1 were able to reproduce the most recent experimentally observed *K*_a_ (11.3 kJ mol^−1 ^^59^) and distance between the Ca and the ligand (0.241–0.254 nm;^60^Table 2), although the distance for Ca–O_carboxylate_ with Set_1 and SPC/fw compared favourably with previously published distances obtained from MD (0.459 nm^21^).

**Table 2:** Free energy of association (Δ*G*_a_), association constant (*K*_a_) and coordination distance (*r*) extracted from free energy profiles in this work and literature experimental and computational values

Ca	Water model	Acronym	Asp			Glu		
			*r*_Ca-OD_ (nm)	−Δ*G*_a_	*K*_a_	*r*_Ca-OD_ (nm)	−Δ*G*_a_	*K*_a_
9	TIP3P	Set_1_TIP3P_	0.274	62.3	7.07 × 10^10^	n.a.	n.a.	n.a.
	SPC/fw	Set_1_SPC/fw_	0.466	4.9	7.07	n.a.	n.a.	n.a.
Based on ref. 39	SPC/fw	Set_2	2.224	18.70	1802.71	n.a.	n.a.	n.a.
Adjusted Set_2	SPC/fw	Set_3_unmodified_	0.238	15.7	548.22	0.242	7.7	22.06
	SPC/fw	Set_3_*σ*+0.5%*σ*_	0.242	10.4	65.46	0.242	6.0	10.98
	SPC/fw	Set_3_*σ*+1.0%*σ*_	0.242	8.1	25.87	0.245	6.3	12.43
	SPC/fw	Set_3_*σ*+2.0%*σ*_	0.245	6.5	13.58	0.248	5.0	7.40
Experiments								
2009^59^				11.3	91.20		7.4	19.05
1990^62^				11.4	97.50			
1987^63^				6.5	13.64			
2012^a^^61^			0.243–0.261					
1998^60^			0.241–0.254					
Simulations								
DFT^22^			0.245			0.243		
2012 MD-CHARMM^61^			0.244–0.260	12.2	133.10			
2017 MD-AMBER-Buck^21^			0.459	4.5	6.07			
Modified CHARMM^28^							6.1	11.54

a No experimental reference.

To improve the energy of association we replaced the Ca parameters with the parameters used in the CaCO_3_ force field (Set_2); we obtained a Δ*G*_a_ of −18.70 kJ mol^−1^, which is in better agreement with experimental values than those from Set_1 (Table 2), although it still overestimates the free energy of association by approximately 7.4 kJ mol^−1^.^22,59–61^ In the free energy profile of Set_2, we observed three distinct minima: around 0.28, 0.33 and 0.50 nm (Fig. 1a), corresponding to biCIP, monoCIP, and SIP, respectively, and in agreement with ref. 28 A less distinguishable minimum was observed around 0.8 nm that correlates with SSIP. The deepest minimum of the energy profile was found at the second well at 0.33 nm. The coordination distance (0.224 nm) is smaller than previously published distances (based on DFT or experimental studies). The free energy of association obtained from the Set_3 simulation was calculated to be −15.7 kJ mol^−1^ and the locations of the different ion pair geometries remained unaltered compared to Set_2. The coordination distance was closer to the distances reported in the literature than those produced by Set_1_TIP3P_, Set_1_SPC/fw_ and Set_2 interactions (Table 2) but slightly shorter than reported by ref. 22, 60 and 61. Thus, the simulations that used the parameters from Set_3 improved the coordination distance and association energy.^59,61^

Subsequently, we optimized the Ca–O_carboxylate_ interaction through modifying the *σ*_Ca-OD_ values in the LJ potential by increasing it by 0.5, 1.0 or 2.0% (see Table 3 for the absolute values), *i.e.* comparable to other studies using the same method for glutamic acid, γ-carboxyglutamic acid (CHARMM).^28^ The energy profile and corresponding Δ*G*_a_ and *K*_a_ (Fig. 1a and Table 2) revealed that a modified *σ* of +0.5% yielded the best match with the latest experimental data.^59^ Moreover, the equilibrium coordination distance was almost the same as published using *ab initio* calculations.^22,61^ Higher increases to *σ* resulted in a lower Δ*G*_a_, although still within the range of experimentally observed values. Increasing *σ* by 2.0% resulted in a distance exactly matching previously reported values,^22^ but the association free energy became slightly lower than the lowest experimental value.^63^ For all modifications, three distinct minima were observed, corresponding to the different ion pair configurations biCIP, monoCIP and SIP. However, increasing *σ* changed the difference between biCIP and monoCIP, especially at *σ* + 0.5%, which reduced this difference the most, from 4.8 kJ mol^−1^ in the unmodified profile to 1.2 kJ mol^−1^. As for Set_3_unmodified_, the modified interactions generated the lowest minimum for monoCIP.

**Table 3:** Absolute heteroatomic Lennard-Jones *σ* values for the Ca^2+^–O_carboxylate_ pair for the combined Set_1 and Set_3 force-field for the Asp/Glu–Ca^2+^ systems

Ca-OD	*σ* (Å)
Set_3_unmodified_	3.10356595082234
Set_3_*σ*+0.5%*σ*_	3.11908378057645
Set_3_*σ*+1.0%*σ*_	3.13460161033056
Set_3_*σ*+2.0%*σ*_	3.16563726983879

As to glutamic acid, a similar approach yielded the energy profiles shown in Fig. 1b. We observed four minima around 0.26, 0.33, 0.61 and 0.74 nm. The first minimum is found to be the deepest and corresponded to the biCIP. Following the same modification method as for aspartic acid, upon increasing *σ* the CIP well became shallower and the separation of SIP and SSIP became less clear. For glutamic acid, the association energies were lower compared to aspartic acid. When comparing to previous studies, the unmodified interaction slightly overestimated the association energy,^59^ while the association energy calculated for *σ* + 0.5% was lower than experimental and computational values.^28,59^ The value of *σ* + 1.0% appeared to achieve a value between the experimental and previous computational association energies. However, since *σ* + 0.5% was closer to the unmodified value and comparable to the more recent MD, we proceed to evaluate the effect on the water structure only for the Set_3_unmodified_ and + 0.5% modification.

### Water structure

**Fig. 2:**
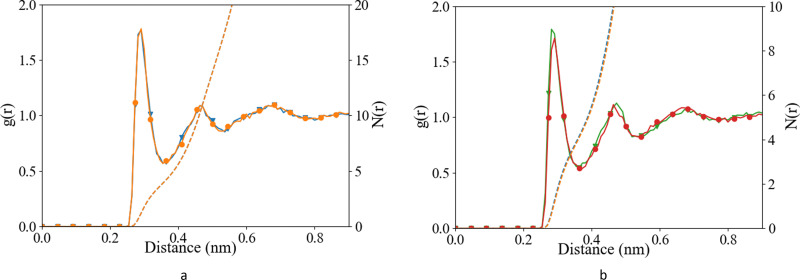
Radial distribution function (after B-spline interpolation) (left axis) and corresponding integral (*N*(*r*)) (right axis) between O_carboxylate_ and O_w_, after 10 ns of classical MD (a) l-Asp, Set_3_unmodified_ (orange; 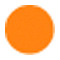), Set_3_*σ*+0.5%*σ*_ (blue; 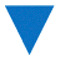), and (b) glutamic acid, Set_3_unmodified_ (red; 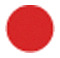), Set_3_*σ*+0.5%*σ*_ (green; 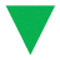) when Ca–O_carboxylate_ were in CIP state.

**Fig. 3:**
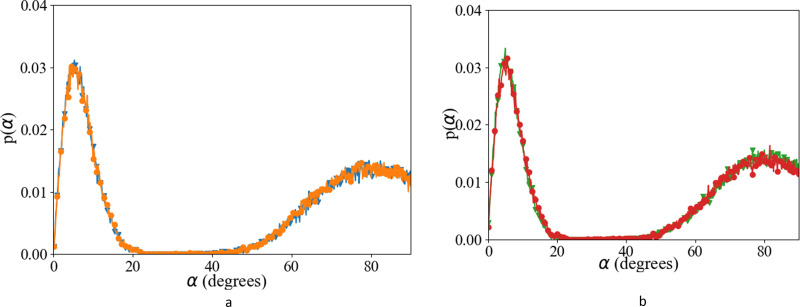
Angular distribution function between O_carboxylate_, O_w_, H_w_ after 10 ns of classical MD (a) l-Asp, Set_3_unmodified_ (orange; 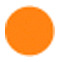), Set_3_*σ*+0.5%*σ*_ (blue; 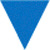), and (b) glutamic acid, Set_3_unmodified_ (red; 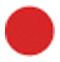), Set_3_*σ*+0.5%*σ*_ (green; 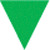) when Ca–O_carboxylate_ were in CIP state.

**Fig. 4:**
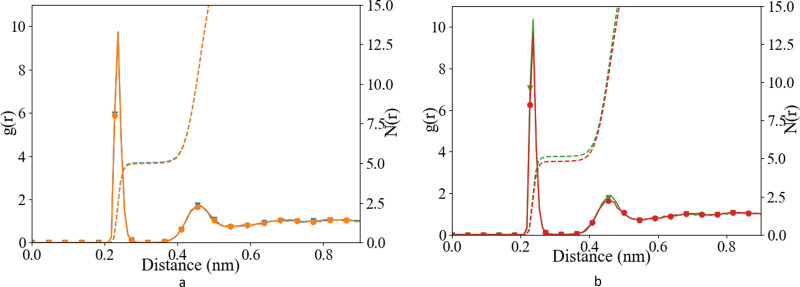
Radial distribution function (after B-spline interpolation) between Ca^2+^ and O_w_, after 10 ns of classical MD (a) l-Asp, Set_3_unmodified_ (orange; 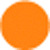), Set_3_*σ*+0.5%*σ*_ (blue; 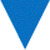), and (b) glutamic acid, Set_3_unmodified_ (red; 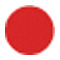), Set_3_*σ*+0.5%*σ*_ (green; 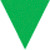) when Ca–O_carboxylate_ were in CIP state.

We have investigated the details of the solvent structuring around the ion pair in the CIP coordination mode. We calculated the radial distribution function (RDF) between the carboxylate-oxygen atom and the water oxygen atom and compared our findings for both the Set_3_unmodified_ and our *σ* + 0.5% modification, as shown in Fig. 2. Two peaks in the RDF were apparent, the first solvation shell at an O_carboxylate_–O_water_ separation of ∼0.29 nm, and a second solvation shell at ∼0.46 nm. The RDF was taken between the O_carboxylate_ and O_water_ with the Ca bonded to the carboxylate. This caused the coordination number to drop and affected the water structuring around the O_carboxylate_, resulting in a sharper second peak similar to previously published RDFs for O_carboxylate_ and O_water_.^28^ The data showed that our modification did not induce a significant difference in these RDFs, for either Asp or Glu. The angular distribution function (ADF) in Fig. 3 confirms that the hydrogen-bond angle between the coordinated water and O_carboxylate_ also remained unaltered upon our modification, when Ca–O_carboxulate_ were in the CIP geometry. To complete our analysis, we studied the RDF between Ca and water when Ca was paired with the biomolecule. Fig. 4 shows that the RDF and coordination of water around calcium also remained the same and in both cases a coordination number of 5 was found. However, note the slight increase in number of water molecules around Ca in CIP with glutamic acid, as the CN increases by 0.33 for Set_3_*σ*+0.5%*σ*_ compared to the unmodified force field.

Overall, the Set_3_unmodified_ interaction in combination with the AMBER description of the amino acid appears to be more realistic than the Set_1 Ca description. However, it still over-estimates the binding of Ca to the carboxylate group as compared with experiment (Table 2). Modifying this single interaction individually to Set_3_*σ*+0.5%*σ*_ helped to improve further the description of the binding energy of the ligand–Ca pair in water (Table 2), without affecting the surrounding water structure (Fig. 2–4).

### Association mechanism

**Fig. 5:**
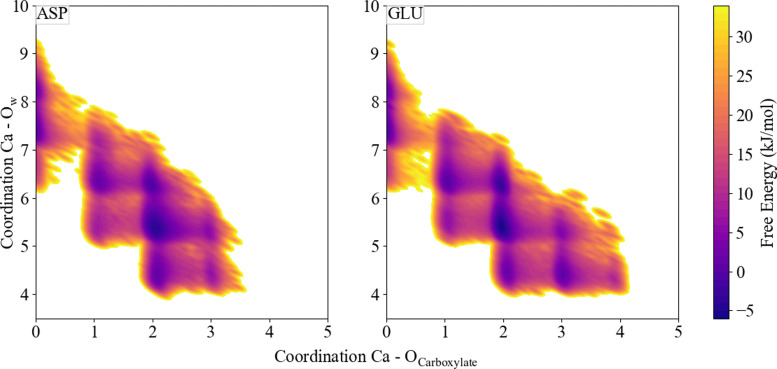
Free energy as a function of distance Ca^2+^ and O_carboxylate_ and coordination number of Ca^2+^ and O_water_.

Insights into the association and dissociation mechanism of Ca^2+^ with the carboxyl group of Asp and Glu were provided by free energy calculations plotted as a function of 2 CV's, in this case the Ca–O_carboxylate_ coordination number and the coordination number of Ca^2+^ to O_water_. The left graph of Fig. 5 shows that in Ca fully dissolved in water the CN_water_ was between ∼6 and ∼9, with three wells indicating three main configurations with CN's around ∼8.3, ∼7.4, and ∼6.6. When Ca was coordinated to one, two or three O_carboxylate_, the CN_water_ ranged from 5 to 8, 4 to 7 and 4 to 6, respectively. The same pattern was observed for Glu, the right-hand side graph of Fig. 5, with the addition of the Ca coordinated to four O_carboxylate_'s. When this latter case was observed the CN_water_ was narrowed to 4.

## ACC formation in the presence of l/d-aspartic acid

Further implications of our proposed modification of the interatomic potential between Ca^2+^ and O_carboxylate_ (Set_3_*σ*+0.5%*σ*_) were investigated in a more complex system. We studied the interaction of freely dissolved Ca^2+^ and CO_3_^2−^ with d-aspartic acid or l-aspartic acid in a system that was highly supersaturated with respect to ACC. We compared cluster structure, cluster density, cluster composition and cluster size. To reproduce the results, we simulated four different starting configurations with a similarity coefficient of less than 70%. Unless stated otherwise, the data shown are therefore the average of the four simulations.

### ACC cluster structure

**Fig. 6:**
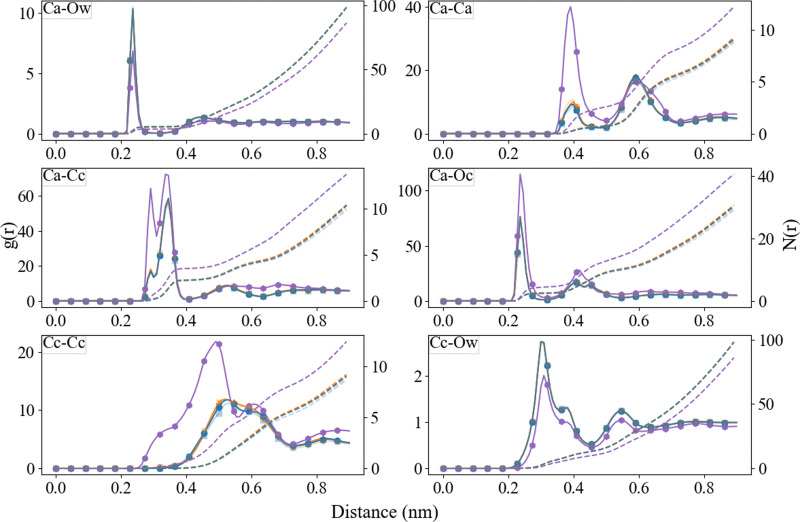
Radial distribution function (after B-spline interpolation) (left axis) and corresponding integral (*N*(*r*)) (right axis) between Ca^2+^, C_c_, O_c_, and O_w_, after 30 ns of simulation. Pure (purple; 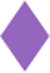), l-Asp with Set_1_SPC/fw_ (transparent blue; 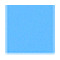), l-Asp Set_3_*σ*+0.5%*σ*_ (blue; 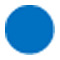), d-Asp Set_1_SPC/fw_ (transparent orange; 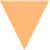), d-Asp Set_3_*σ*+0.5%*σ*_ (orange; 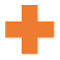).

**Fig. 7:**
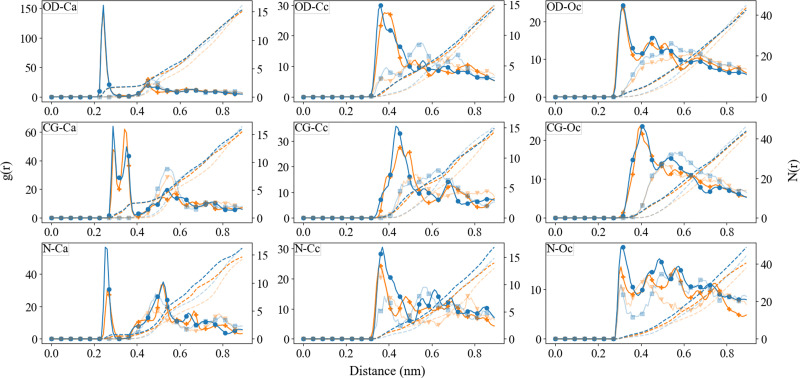
Radial distribution function (after B-spline interpolation) between Ca^2+^, C_c_, O_c_, with the different functional groups in aspartic acid after 30 ns of simulation. l-Asp with Set_1_SPC/fw_ (transparent blue; 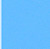), l-Asp Set_3_*σ*+0.5%*σ*_ (blue; 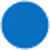), d-Asp Set_1_SPC/fw_ (transparent orange; 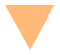), d-Asp Set_3_*σ*+0.5%*σ*_ (orange; 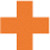). The data from Set_3_*σ*+0.5%*σ*_ was averaged over the four duplicates, Set_1_SPC/fw_ was a single simulation.

The radial distribution functions between different species, obtained from the final 10 ns of the simulations, are reported in Fig. 6 and 7. For comparison, we have also included the RDFs of a single simulation using Set_1_SPC/fw_. As observed in the ACC RDFs, the distances remained unaltered upon the refinement of the force field. The RDFs between the different parts of Asp, as shown in Fig. 7, reveal a clear difference between the force fields in the distance between the carboxyl oxygen (OD) and the coordinated Ca, which decreased from 0.46 nm to 0.24 nm. In addition, a clear separation between biCIP and monoCIP was observed at 0.28 and 0.35 nm, respectively. The intensities of the biCIP and monoCIP for d-Asp were inverted compared to l-Asp. The refined O_carboxylate_–Ca potential parameters also resulted in more structure between the amine group and Ca, as reflected by the more pronounced minima in the N–Ca and especially the N–C_c_ RDFs. Data from the other tested *σ* values (single simulations) can be found in the ESI,† Fig. S2 until Fig. S5).

**Fig. 8:**
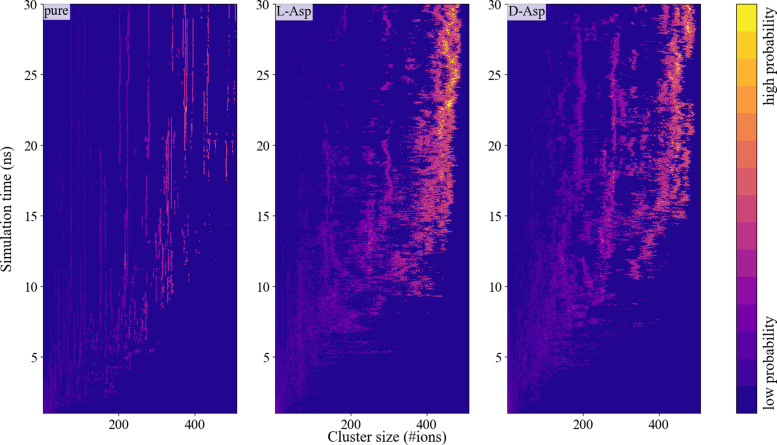
Probability intensity plots of different biomolecule–CaCO_3_-systems showing the probability of an ion to be in a cluster with a certain size as a function of time (averaged over four simulations).

### ACC cluster density

**Fig. 9:**
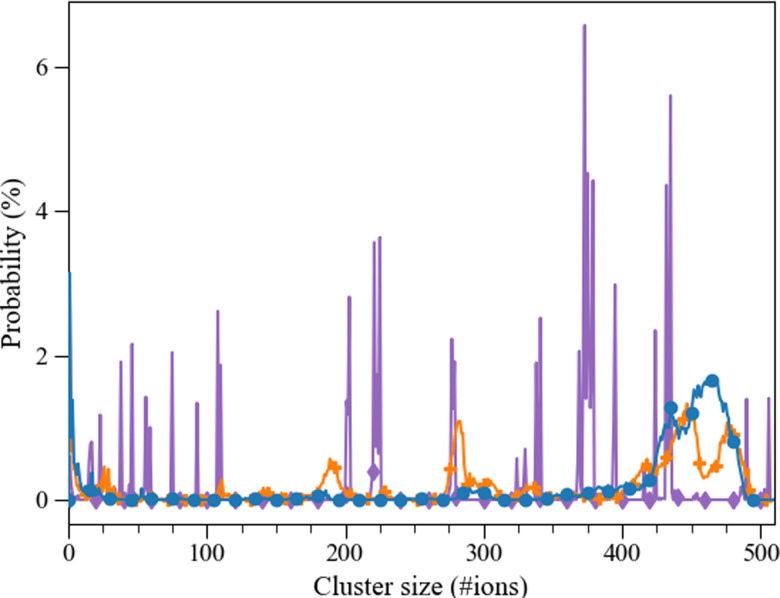
Probability (%) of an aggregate of certain size (consisting of a number of ions) taken from the last 10 ns of the 30 ns simulation. Pure (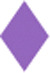), l-Asp Set_3_*σ*+0.5%*σ*_ (blue; 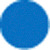), d-Asp Set_3_*σ*+0.5%*σ*_ (orange; 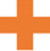).

We calculated the density of CaCO_3_ around different groups in the biomolecule, as well as the average density of the ACC clusters, by calculating the density around every Ca^2+^ in the system (Table 4). The density is calculated around all ions, independent of the cluster they belong to. Fully solvated ions will therefore contribute to the density by lowering it. Since it is a dynamic system, the cluster size of the largest cluster varies. And since the largest cluster contains the most ions this cluster will also contribute the most to the density in Table 4. The cluster size distribution taken from the same part of the simulations as the densities, is shown in Fig. 9. The densities in the pure CaCO_3_ system were consistently ∼0.2 g cm^−3^ higher than in the biomolecule systems. An increase in density around the functional groups of the biomolecule, due to the new interaction parameters (Set_3_*σ*+0.5%*σ*_), was most noticeable for d-Asp, as well as the average ACC density. In contrast, the measured densities in both l-Asp and the formed ACC were less affected by the modified interaction. The difference between l-Asp and d-Asp was small and, due to the higher variability within the duplicates, and the densities around the functional groups of the two systems, were indistinguishable. The densities around the O_carboxyl_ were slightly higher than values reported in the literature, although note that those previously reported values were for amino acid groups within a protein.^4^ All formed clusters had lower densities compared to hydrated and ACC clusters determined by ref. 21 In the latter, the ACC clusters were generated prior to the simulations, *via* a melting process of randomly packed molecules with a restricted number of water molecules. Therefore, the clusters reported before formed *via* a different pathway and the initial cluster formation steps were not part of their simulations.^21^ This may be an explanation for the difference in density/water content.

**Table 4:** Density of Ca^2+^ and CO_3_^2−^ ions around the functional groups of the biomolecules and around Ca^2+^ obtained from the last 10 ns after 30 ns of simulation

Density (g cm^−3^)			
System	O_Carboxyl group_	N_Amine_	Ca^2+^
Pure	n.a.	n.a.	0.73 ± 0.023
Set_3_*σ*+0.5%*σ*_			
l-Asp	0.81 ± 0.071	0.92 ± 0.127	0.55 ± 0.030
d-Asp	0.82 ± 0.172	0.78 ± 0.180	0.56 ± 0.007
Set_1_SPC/fw_			
l-Asp	0.78	0.83	0.46
d-Asp	0.58	0.58	0.47
Literature			
l-Asp^21^			∼1.1^a^–1.4
Aspartate in lysozyme^4^	∼0.71		

a Hydrated ACC.

### ACC cluster size distribution and their solvent accessible surface area

The calcium carbonate ions in the systems with freely dissolved ions, as well as those with one l/d-aspartic acid, aggregated over the course of the simulation. In all simulations, the ions formed clusters, and in all systems with a biomolecule, the biomolecule was found incorporated in the largest cluster (except for one of the d-Asp simulations). The agglomeration was followed *via* a calculation of the cluster size distribution at every picosecond of simulation time (Fig. 8). Both l-Asp and d-Asp systems revealed a different clustering pattern compared to pure ACC. Whereas the system without the biomolecule showed sharp and constant lines throughout the simulation, the systems with the biomolecule showed a more smeared out pattern, revealing that the clusters varied more in cluster size through constant addition of ions or small clusters. In contrast, the cluster size variation in the pure ACC is most likely due to the aggregation of various clusters.

The average cluster size distribution over the final 10 ns, Fig. 9, shows that the largest cluster in the pure system contained ±507 ions, which is somewhat higher than the l-Asp (±456 ions) and d-Asp (±478 ions) systems. The pure ACC system also comprised more intermediate clusters with a constant size and a longer lifetime, whereas similar intermediate cluster sizes of ±191 and ±284 ions were found in the D-Asp system (Fig. 9), although these intermediate cluster sizes were not observed in the l-Asp system (Fig. 9). If we average the data over the final 10 ns, we see the same contrast between the pure and amino acid-containing systems, with wider peaks in the cluster size distributions when the biomolecule is present (Fig. 9); note that this is also an average of four different simulations. Another difference between the pure CaCO_3_ system and the systems with l- or d-Asp was the time it took to observe the largest aggregates. While for l- and d-Asp the largest aggregate was already detected before 15 ns, in the pure system it took above 20 ns to form the largest aggregate (Fig. 8).

**Fig. 10:**
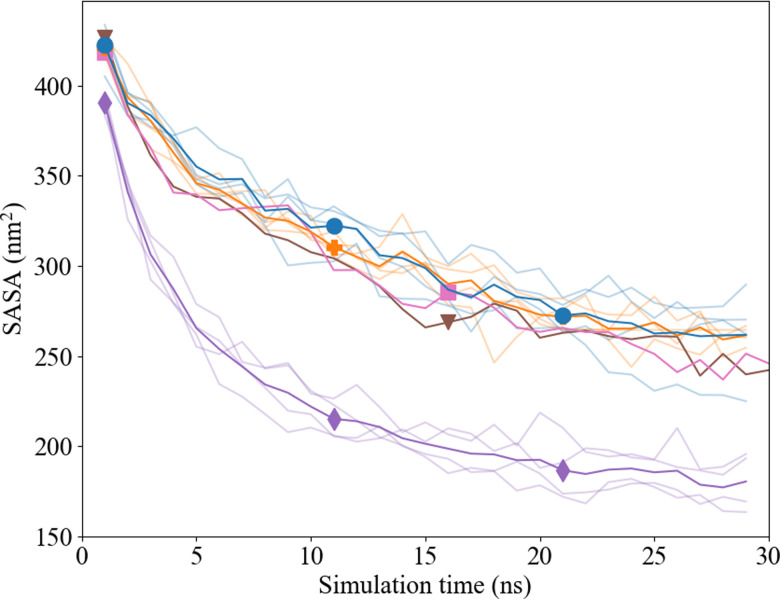
SASA analysis using the Shrake and Rupley algorithm of Pure (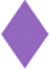), l-Asp with Set_1_SPC/fw_ (pink; 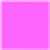), l-Asp Set_3_*σ*+0.5%*σ*_ (blue; 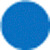), d-Asp Set_1_SPC/fw_ (brown; 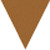), d-Asp Set_3_*σ*+0.5%*σ*_ (orange; 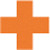), the transparent lines represent the individual calculations.

The solvent accessible surface area (SASA) of the Ca^2+^ and CO_3_^2−^ ions in solution was followed over time to study the effect of clustering on the surface area that was exposed to water with the new proposed interaction parameters. The volume within the cluster was assumed accessible and/or occupied by water molecules when it was equal to or bigger than the volume of one water molecule (with a radius of 0.14 nm). In the presence of Asp, the total SASA decreases by ∼40% within 30 ns, where we observed no difference between the d- and l-Asp systems (Fig. 10). However, a clear difference was seen in comparison with the pure system, where the SASA for the pure systems shrank significantly more over time (∼54%), leaving a much dryer cluster.

## Implications

### Interaction of calcium with carboxyl groups in aspartic and glutamic acid

In the past years, several studies have emphasised the importance of a re-evaluation of the parametrization of the interactions of charged amino acid side chains with water and ions to achieve a more accurate description of these systems.^15,43,44^ In previous simulations using AMBER-based force field parameters,^21^ the conclusion was drawn that it is mainly the amine group that interacts with the CO_3_^2−^ or an ACC surface rather than the carboxyl group, even though, based on experimental evidence, the latter was expected to be the main interacting functional group. The current study into the carboxyl-oxygen–calcium interaction revealed that parameter Set_1 has one of the highest repulsion terms in the Lennard-Jones potential, and although the attraction term is also higher, the overall result is a stronger repulsion in the other force field parameters,^50–58^ with implications for the simulation of single amino acids and larger biomolecules that are rich in aspartic and/or glutamic acid.^64,65^ Another description of Ca, derived from a refined CaCO_3_–water_SPC/fw_ force field,^39^ with the lowest repulsion and attraction values, also has a repulsion/attraction ratio of the same order of magnitude as the Ca in Set_1.

A common water model used in simulations with biomolecules is the TIP3P water model.^66–70^ However, the energy profile obtained for the Ca description from Set_1 with TIP3P water overestimates the association constant of Ca with both amino acids by nine orders of magnitude, as seen before in AMBER- and CHARMM-based force fields.^28,71^ This discrepancy is mainly due to a lack of well-defined, explicit parameterization of the interaction between the amino acid and calcium,^28^ although this interaction is able to reproduce the different configurations upon association.^47^ Unfortunately, the TIP3P model has been shown to have a self-diffusion coefficient for water that is twice the experimental value.^40^ Moreover, other water models are considered to be more suitable for CaCO_3_ systems in terms of water dynamics and water structuring.^40,72,73^ For this reason, we have combined the parameters from Set_1 with SPC/fw water. With this force field, we obtained an energy profile that agreed closely with previously published results,^21^ although these authors used a Buckingham potential rather than the Lennard Jones potential employed in this work, to describe the interaction between Ca and the biomolecule. A Buckingham potential is wider, leading to a less structured solvation shell,^74^ which therefore contributes to the difference of −0.4 kJ mol^−1^ between their^21^ and our results (Table 2). However, unlike the energy profile of Set_1_TIP3P_, the Set_1_SPC/fw_ force field is unable to capture the structural configurations of the ion pair and shows a global minimum at ∼0.466 nm, indicating that the SIP configuration would be preferred over CIP. Nevertheless, Set_1_SPC/fw_ shows overall improved −Δ*G*_a_ (Table 2), and in the current work, we therefore refined this force field to capture the carboxyl-Ca configuration known from experiments.

With the refined force field Set_3_*σ*+0.5%*σ*_ for aspartic acid, the distance between the carboxyl-oxygen and the calcium corresponds to the monoCIP structure, although the difference between monoCIP and biCIP minima, at 1.22 kJ mol^−1^ compared to 4.79 kJ for Set_3_unmodified_ is the smallest. In terms of stability, this means that a monoCIP configuration is favoured over a biCIP, which agrees with experimental observations using ^13^C nuclear magnetic resonance (NMR).^75^ Moreover, the water structure, in terms of RDF and hydrogen bond-angle around the O_carboxylate_ compares well with those previously reported for O_carboxylate_ in acetate.^44^ In short, the refined force field captures known interaction energies and structures for calcium coordinating to the carboxyl group of aspartic acid.

For glutamic acid, the association energy is lower than for aspartic acid: due to the extra CH_2_ group, the charge density in Glu is lower, and therefore the attraction of Ca^2+^ is weaker. Although the unmodified interaction gives a Δ*G*_a_ that compares better to values obtained with an unmodified CHARMM-based force field,^28^ it is still slightly overestimated compared to experimental observations^59^ and refined computational results.^28^ In all cases the biCIP is more stable compared to monoCIP, in contrast with aspartic acid but in agreement with other MD simulations for Ca–Glu interactions.^28^

In the first step towards association of a dissolved calcium ion with another (dissolved) biomolecule such as Asp or Glu, Ca must lose one of its ∼8.3 water molecules. The first transition state can be characterized by a coordination number of 7.4. The most probable mechanism for this first step is that a water molecule leaves the solvation shell before Ca can approach more closely. Following the energy landscape, one of the O_carboxylate_'s coordinates to Ca before another water molecule can leave the coordination shell. A similar mechanism was observed upon increasing the O_carboxylate_ CN further. All three exchange mechanisms – associative, dissociative and interchange – as previously described,^76^ are possible Ca association mechanisms and observed here for Asp and Glu (Fig. 5). However, the lowest energy pathway reveals a preference for an associative mechanism, considering the darkest shaded coordination states in the energy landscape in Fig. 5. This associative mechanism can be traced by following the local minima in the free energy landscape, *via* ∼7O_water_ and 1O_carboxylate_, ∼6O_water_ and 1O_carboxylate_, ∼6O_water_ and 2O_carboxylate_ to ∼5O_water_ and 2O_carboxylate_. Due to the longer chain length in Glu, the molecule is more flexible making it possible to coordinate all four oxygens to Ca, however this is not the most energetically favoured configuration. Both, Asp and Glu, agree with Ca^2+^–ligand exchange mechanisms identified with multidimensional vibrational spectroscopy using different ligands.^77^

Based on the above, we consider that the refined parameters to describe the interaction of calcium with a biomolecule are suitable to be used to study more complex systems.

### Implications for ACC cluster formation in the presence and absence of aspartic acids

Due to the extremely high concentration of calcium and carbonate in solution (0.89 M), we could observe rapid cluster formation within MD-accessible timescales. However, this high concentration also means that the starting solution is not stable and will undergo a spontaneous phase separation, *via* spinodal decomposition.^78^ Formation of clusters will occur without any free energy barrier to overcome and without a critical nucleus size. This observation is in agreement with experiments using a slightly lower concentration of 0.50 M CaCO_3_^79^ and even lower concentrations (0.05 M),^80,81^ and with previous MD (0.53 M),^82^ and other theoretical results.^83,84^

In our simulations, the rapid formation of the initial ion pairs and small clusters is followed by aggregation of ion pairs and small clusters into larger clusters and – in the presence of biomolecules – almost always the incorporation of the biomolecule into the largest cluster. Further growth of the clusters is substantially slower, because the number of free ions, ion pairs and clusters in solution decreases and, consequently, the agglomeration frequency goes down. Moreover, the lower diffusivity of the larger clusters slows down further growth and makes the agglomeration process and formation of ACC more diffusion-limited.^85^

The simulation of ACC cluster formation in the presence of d- and l-Asp shows some clear differences compared to the cluster formation in the absence of a biomolecule. Overall, with the refined interactions (Set_3_*σ*+0.5%*σ*_), the aspartic acid enantiomers have the same impact on ACC cluster formation and the cluster stabilization as observed for Set_1_SPC/fw_ and in previous computational^86^ and experimental work.^64^ Compared to the pure system, ACC clusters in the presence of Asp formed more rapidly (after 15 ns instead of 20 ns, respectively) and were on average slightly larger. This minimal effect on cluster size is in agreement with findings in experiments, where only marginal effects were reported for the effect of aspartic acid monomers on the particle size.^87^ Furthermore, the aggregates remained more hydrated, which resulted in a more flexible cluster size (*i.e.*, constant rearrangement of the aggregate including hydration and dehydration processes). The stabilization of the hydrated clusters could explain the inhibition effects of Asp on calcite growth as observed in experiments.^88^

In terms of cluster size distribution, development in time and hydration levels, both enantiomers behave similarly with more abundant intermediate cluster sizes in the presence of d-Asp. The only clear difference we could identify between the two enantiomers was in terms of ion structuring around the functional groups of the biomolecule. The refined and stronger attraction of Ca with the carboxyl groups resulted in clear changes in RDFs between Ca and the functional groups of the two enantiomers, with a difference in preference for bi- and monoCIP changes between l and d. The biCIP configuration is preferred of over the monoCIP configuration in the case of l-Asp, whereas the monoCIP configuration is preferred in the presence of d-Asp, as it is in the simulations of single calcium ions with l-Asp. This difference could be of importance during other stages of crystal formation, but during the formation of the clusters as part of the spinodal decomposition, the chirality of Asp has an insignificant impact.

In addition to the stabilization of hydrated clusters, other implications of the insights presented in this work for biomineralization can, for example, be related to the role of biomolecules in combination with impurities like magnesium cations. Previous studies have shown that the presence of biomolecules accelerate the growth rate of a calcite surface,^89^ but where impurity uptake in the crystal is concerned, it has been found that Mg is more strongly affected by the biomolecule compared to Ca.^89^ Our simulations showed that l- and d-aspartic acid both have a stabilizing effect on the formation of hydrated ACC and could therefore provide a rather fluid-like/malleable reservoir of calcium and carbonate that can facilitate a fast transformation and/or fast ion-attachment and growth of the crystal.

## Conclusion

In this study we have employed classical molecular dynamics simulations, using a number of refined force fields, to investigate the interaction of Ca with aspartic and glutamic acid. The results show that the ability to describe the association of the ions and the different structural stages upon ion pairing is crucially affected by the potential parameters, which therefore need to be chosen carefully. Using previously published parameters (Set_1^9^), the force field does not reproduce the correct association energy (when combined with the TIP3P water model), nor the correct structural details (when combined with the SPC/fw model). The refined interaction parameters proposed here adequately enhanced the binding distance and the Δ*G*_association_ without disrupting the solvent structure around the biomolecule and the ion. This new description shows a strong impact in more complex systems, where Ca-binding is now favoured over CO_3_^2−^, in contrast to previously reported computational results, but supporting experimental findings. Therefore, we would recommend using the Set_3_*σ*+0.5%*σ*_ interactions for Ca–O_carboxylate_ when conducting MD of systems including Ca^2+^ and AMBER-based biomolecules with carboxyl functional groups.

Subsequently, the impact of aspartic acid on the formation of ACC *via* spinodal decomposition was studied by performing MD simulations of solutions with dissolved calcium and carbonate ions in the absence and presence of d- or l-Asp. Both enantiomers had a similar effect on the aggregation of the clusters, where the biomolecule was incorporated in the largest cluster and the aggregates retained more water. As a result, the hydrated ACC was more malleable. In our case, an underestimation of the association constant (Set_1_SPc/fw_) led to similar stabilization of the hydrated ACC. Despite the difference in internal structure of ACC around the functional groups of Asp. In other systems with higher biomolecule concentrations and/or lower mineral concentrations (out of the spinodal decomposition regime), the effect of the Ca-trapping will probably be more significant and more easily compared to experimental observations of calcium trapping mechanisms.^23^

In general, our study has shown that aspartic acid monomers can be useful tools in biomineralization processes, for example to increase the hydration of amorphous calcium carbonate (ACC), thereby inhibiting dehydration. Potentially, these monomers can also be used to tailor calcium carbonate formation including in the reduction of scale formation.

## Author contributions

Conceptualization, J. A. K., S. E. R. H. and M. W.; methodology, J. A. K. and S. E. R. H.; validation, J. A. K., S. E. R. H. and M. W.; formal analysis, J. A. K. and S. E. R. H.; investigation, J. A. K., S. E. R. H. and M. W.; resources, M. W.; data curation, J. A. K.; writing—original draft preparation, J. A. K.; writing—review and editing, S. E. R. H. and M. W.; visualization, J. A. K.; formal analysis and review N. H. L. supervision, M. W.; project administration, M. W.; funding acquisition, M. W. All authors have read and agreed to the published version of the manuscript.

## Conflicts of interest

The authors declare no conflict of interest. The funders had no role in the design of the study; in the collection, analyses, or interpretation of data; in the writing of the manuscript, or in the decision to publish the results.

## Supplementary Material

CP-025-D2CP02879D-s001
