# Supplementary material for: Recalibrating the calcium trap in amino acid carboxyl groups via classical molecular dynamics simulations
Source: Phys Chem Chem Phys. 2022 Dec 5;25(2):1220–35. doi: 10.1039/d2cp02879d (PMC9811642; doi:10.1039/d2cp02879d)
Supplement: CP-025-D2CP02879D-s001 [file CP-025-D2CP02879D-s001.pdf]

Electronic Supplementary Material (ESI) for Physical Chemistry Chemical Physics.
This journal is © the Owner Societies 2022

# Supplementary Information: Recalibrating the calcium trap in amino acid carboxyl groups via classical molecular dynamics simulations

Janou A. Koskamp[1], Sergio E. Ruiz Hernandez[1], Nora H. de Leeuw[1,2] and Mariette Wolthers[1*]

[a. 1] *Department of Earth Sciences, Utrecht University, 3584 CB Utrecht, The Netherlands; j.a.koskamp@uu.nl (J.A.K.); s.e.ruizhernandez@uu.nl (S.E.R.H.); n.h.deleeuw@uu.nl (N.H.d.L.)*
[b. 2] *School of Chemistry, University of Leeds, Leeds LS2 9JT, UK; n.h.deleeuw@leeds.ac.uk (N.H.d.L.)*
[c. *] *Correspondence: m.wolthers@uu.nl; Tel.: +31302535042*

## Lennard-Jones potentials Ca – Aspartic Acid

The intermolecular Lennard-Jones potentials between Ca and Aspartic Acid according to the different calcium potential parameters, Set_1, Set_2, and Set_3 as mentioned in Table 1 in the main text.

Table S1 Lennard-Jones potential parameters Ca - Aspartic acid Set_1

| Atom i | Atom j | $\varepsilon_{ij}$ eV | $\sigma_{ij}$ (Å) |
|---|---|---|---|
| Ca | N | 0.01212336177754750 | 3.33809926201551 |
| Ca | H1 | 0.00368424682452194 | 2.24763923086026 |
| Ca | CT | 0.00972539310852265 | 3.41293475422537 |
| Ca | H2 | 0.00368424682452194 | 2.69308858950367 |
| Ca | H3 | 0.00368424682452194 | 3.03786639363011 |
| Ca | C2 | 0.00862279272582844 | 3.41293475397241 |
| Ca | OD | 0.01347436327552800 | 3.19306095082234 |

Table S2 Lennard-Jones potential parameters Ca - Aspartic acid Set_2

| Atom i | Atom j | $\varepsilon_{ij}$ eV | $\sigma_{ij}$ (Å) |
|---|---|---|---|
| Ca | N | 0.000993522376406289 | 3.39225326201551 |
| Ca | H1 | 0.000301927941072037 | 2.30179323086026 |
| Ca | CT | 0.000797006296599970 | 3.46708875422537 |
| Ca | H2 | 0.000301927941072037 | 2.74724258950367 |
| Ca | H3 | 0.000301927941072037 | 3.09202039363011 |
| Ca | C2 | 0.000706647023937694 | 3.46708875397241 |
| Ca | OD | 0.001104238384344610 | 3.24721495082234 |

Table S3 Lennard-Jones potential parameters Ca - Aspartic acid Set_3

| Atom i | Atom j | $\varepsilon_{ij}$ eV | $\sigma_{ij}$ (Å) |
|---|---|---|---|
| Ca | N | 0.00285309254151 | 3.24860426201551 |
| Ca | H1 | 0.00086704474625 | 2.15814423086026 |
| Ca | CT | 0.00228875843601 | 3.32343975422537 |
| Ca | H2 | 0.00086704474625 | 2.60359358950367 |
| Ca | H3 | 0.00086704474625 | 2.94837139363011 |
| Ca | C2 | 0.00202927422809 | 3.32343975397241 |
| Ca | OD | 0.00317103507000 | 3.10356595082234 |

## Supplementary Graphs and Figures

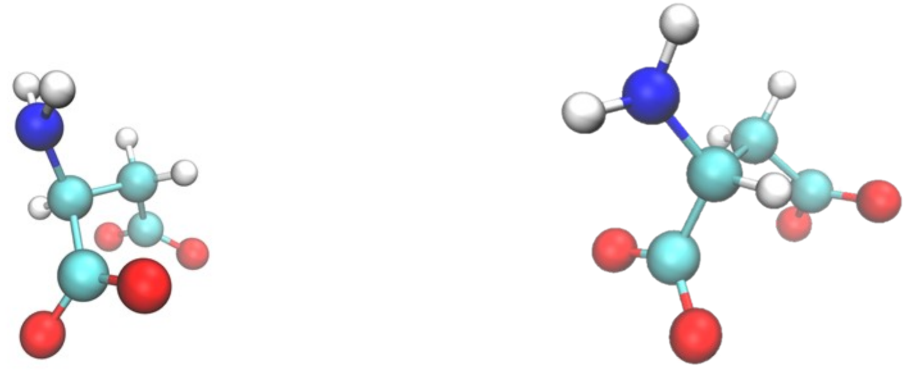

**D-aspartic acid** **L-aspartic acid**

Figure S1 Molecular structure of the studied biomolecules. Nitrogen (blue), Carbon (cyan), Oxygen (red) and Hydrogen (white).

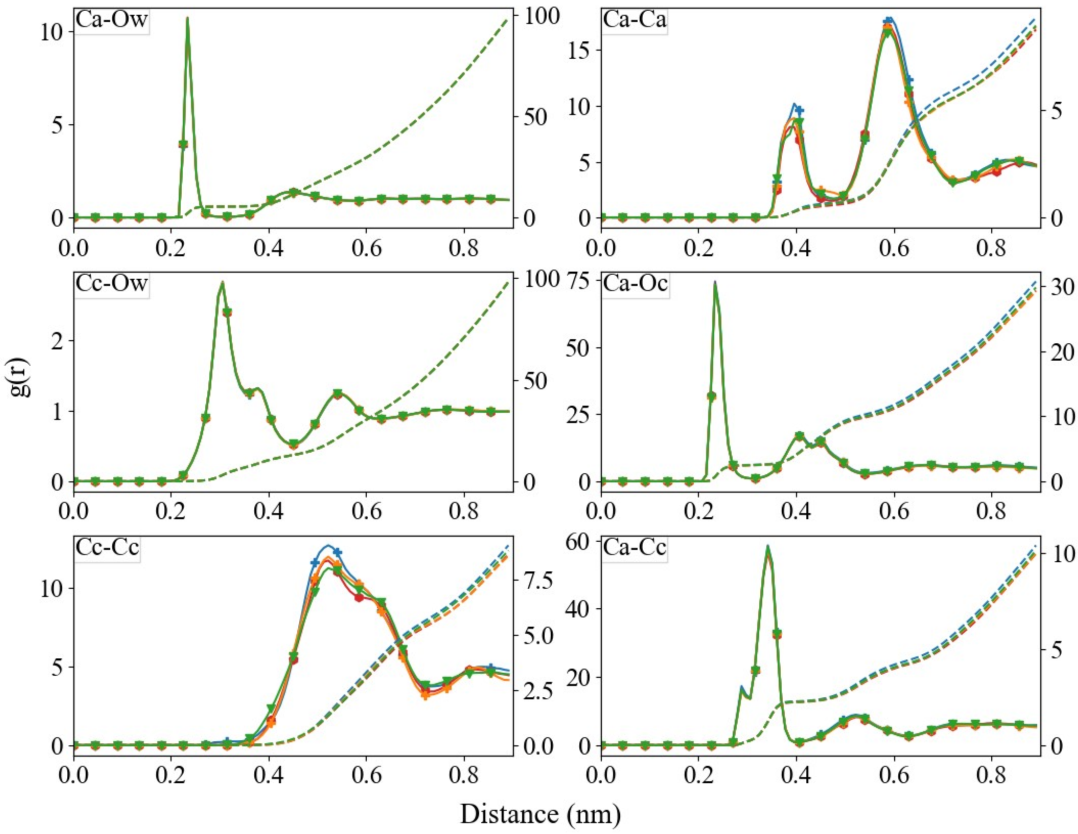


Figure S2 Radial distribution function (left axis) and corresponding integral (N(r)) (right axis) between $Ca^{2+}$, $C_c$, $O_c$, and $O_w$, after 30 ns of simulation. **D-Asp** using different forcefields**;** Set_3$_{unmodified}$ (red; ⬢), Set_3$_{\sigma+0.5\%\sigma}$ (blue; ✚), Set_3$_{\sigma+1.0\%\sigma}$ (orange; +), and Set_3$_{\sigma+2.0\%\sigma}$ (green; ▼)

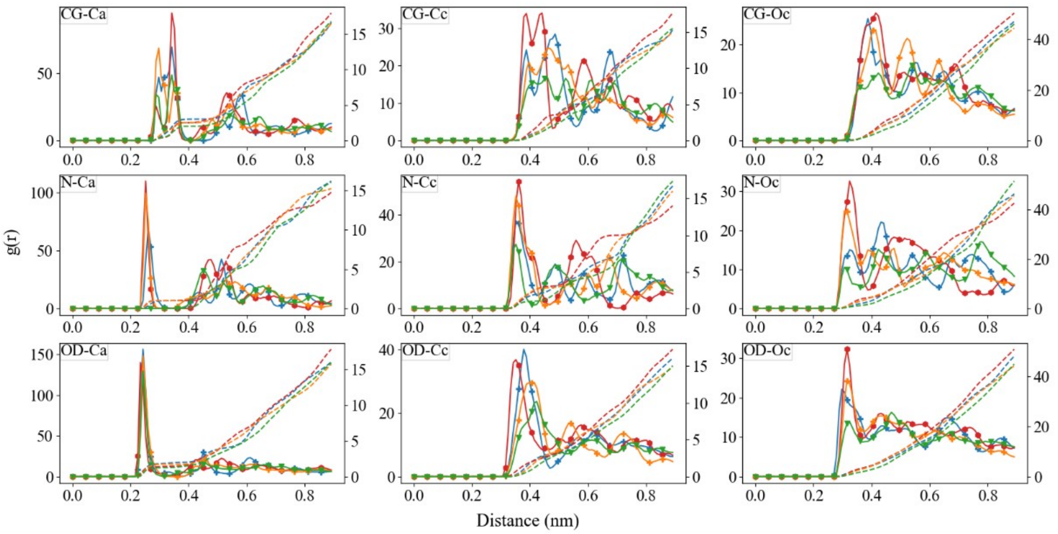


Figure S3 Radial distribution function between $Ca^{2+}$, $C_c$, $O_c$, with the different functional groups in aspartic acid after 30 ns of simulation. **D-Asp** using different forcefields**;** Set_3$_{unmodified}$ (red; ⬢), Set_3$_{\sigma+0.5\%\sigma}$ (blue; ✚), Set_3$_{\sigma+1.0\%\sigma}$ (orange; +), and Set_3$_{\sigma+2.0\%\sigma}$ (green; ▼)

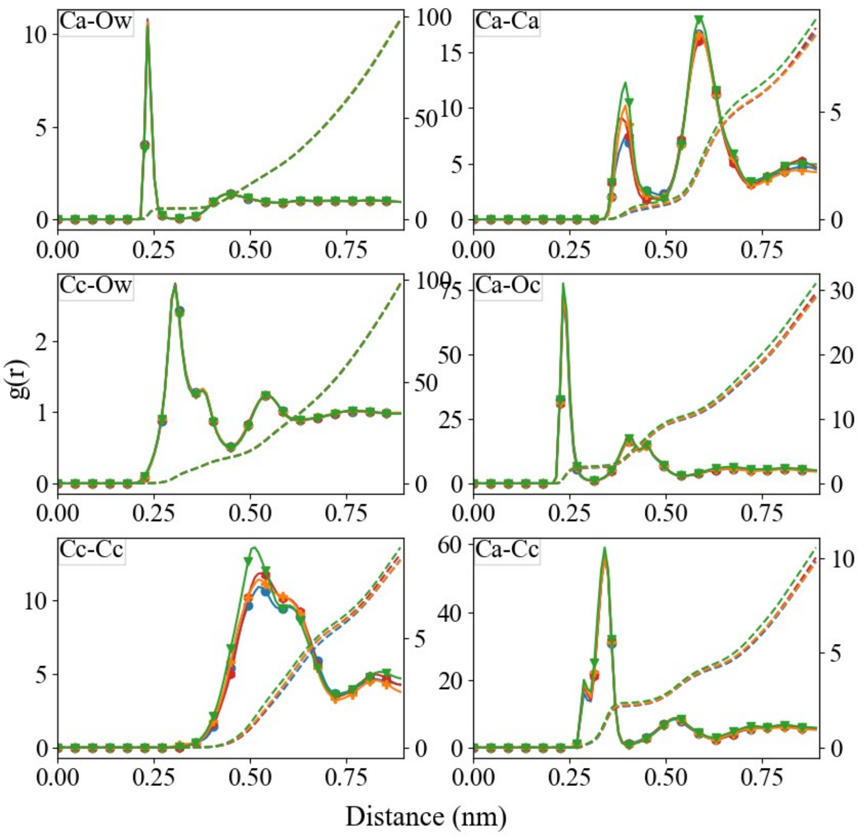


Figure S 4 Radial distribution function (left axis) and corresponding integral (N(r)) (right axis) between $Ca^{2+}$, $C_c$, $O_c$, and $O_w$, after 30 ns of simulation. **L-Asp** using different forcefields**;** Set_3$_{unmodified}$ (red; ⬢), Set_3$_{\sigma+0.5\%\sigma\sigma+0.5\%}$ (blue; ✚), Set_3$_{\sigma+1.0\%\sigma}$ (orange; +), and Set_3$_{\sigma+2.0\%\sigma}$ (green; ▼)

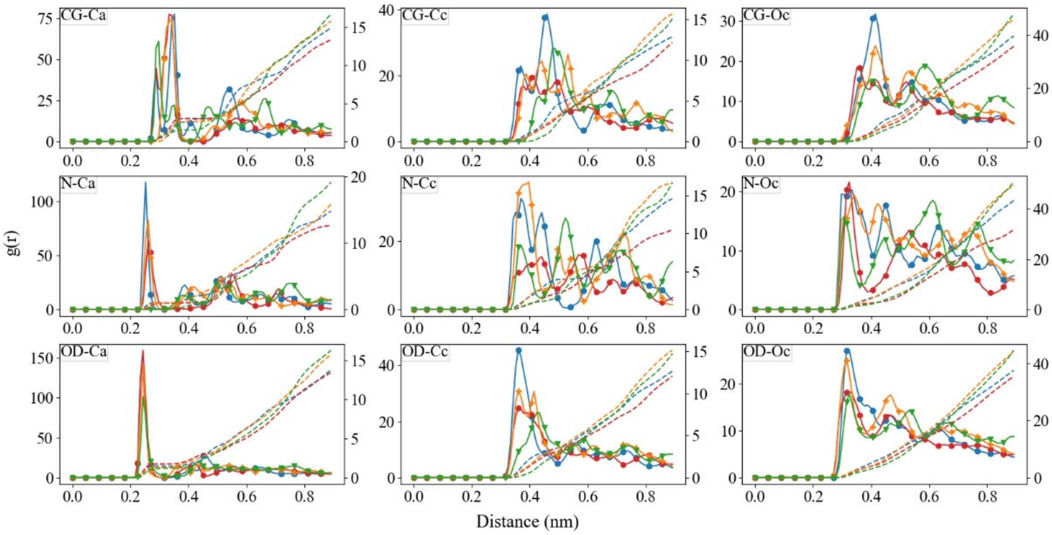


Figure S 5 Radial distribution function between $Ca^{2+}$, $C_c$, $O_c$, with the different functional groups in aspartic acid after 30 ns of simulation. **L-Asp** using different forcefields**;** Set_3$_{unmodified}$ (red; ⬢), Set_3$_{\sigma+0.5\%\sigma}$ (blue; ✚), Set_3$_{\sigma+1.0\%\sigma}$ (orange; +), and Set_3$_{\sigma+2.0\%\sigma}$ (green; ▼)
